# Charge Mediated Compaction and Rearrangement of Gas-Phase Proteins: A Case Study Considering Two Proteins at Opposing Ends of the Structure-Disorder Continuum

**DOI:** 10.1007/s13361-017-1692-1

**Published:** 2017-06-05

**Authors:** Jacquelyn R. Jhingree, Bruno Bellina, Kamila J. Pacholarz, Perdita E. Barran

**Affiliations:** Manchester Institute of Biotechnology, University of Manchester, 131 Princess Street, Manchester, M1 7DN UK

**Keywords:** Ion mobility mass spectrometry, ETD, ETnoD, Effect of charge on protein structure

## Abstract

Charge reduction in the gas phase provides a direct means of manipulating protein charge state, and when coupled to ion mobility mass spectrometry (IM-MS), it is possible to monitor the effect of charge on protein conformation in the absence of solution. Use of the electron transfer reagent 1,3-dicyanobenzene, coupled with IM-MS, allows us to monitor the effect of charge reduction on the conformation of two proteins deliberately chosen from opposite sides of the order to disorder continuum: bovine pancreatic trypsin inhibitor (BPTI) and beta casein. The ordered BPTI presents compact conformers for each of three charge states accompanied by narrow collision cross-section distributions (^TW^CCSD_N2→He_). Upon reduction of BPTI, irrespective of precursor charge state, the ^TW^CCS_N2→He_ decreases to a similar distribution as found for the nESI generated ion of identical charge. The behavior of beta casein upon charge reduction is more complex. It presents over a wide charge state range (9–28), and intermediate charge states (13–18) have broad ^TW^CCSD_N2→He_ with multiple conformations, where both compaction and rearrangement are seen. Further, we see that the ^TW^CCSD_N2→He_ of the latter charge states are even affected by the presence of radical anions. Overall, we conclude that the flexible nature of some proteins result in broad conformational distributions comprised of many families, even for single charge states, and the barrier between different states can be easily overcome by an alteration of the net charge.

**Electronic supplementary material:**

The online version of this article (doi:10.1007/s13361-017-1692-1) contains supplementary material, which is available to authorized users.

**Graphical Abstract:**
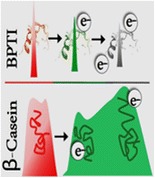
ᅟ

## Introduction

It has become common practice to infer the populations of protein conformations in the gas phase from the charge state distribution (CSD) it presents following electrospray ionization (ESI) [1, 2]. It is apparent that the CSD can be affected by many factors, including but not limited to solution phase structure (charge), ionic strength of solvent, solvent accessible sites (protonation in positive ion mode and deprotonation in negative ion mode), and solution-phase and gas-phase basicity [3–9]. It is also well known that ESI conditions can alter the CSD and by inference the conformational distribution, although there is a clear preference for a particular charge state distribution, especially for structured proteins electrosprayed from salty solutions [10, 11]. Many studies have shown that the more charges a protein possess post-ESI, the more unfolded it is; and for some proteins and some solution conditions this can be attributed to the protein existing in a range of conformational states in solution where a differing number of basic or acidic groups are available for protonation or deprotonation respectively [6, 12, 13]. It is also plausible that the electrospray process causes proton transfer as the protein desolvates. Higher charge states are susceptible to repulsive Coulombic effects caused by proximal charges, which in turn will affect the structure of the gas-phase ion [9, 14].

Ion mobility spectrometry (IMS) is a separation technique that is increasingly used in combination with mass spectrometry (IM-MS) to measure gas-phase conformation of desolvated molecules [15–17]. The mobility of an ion is a measure of the velocity of a given ion in a device filled with a neutral buffer gas under the influence of a weak electric field, where the number of collisions with the gas along with the charge and shape of the ion means the ion elutes as an arrival time distribution, which can be used to determine the rotationally averaged collision cross-section (CCS) of the mass selected ion. A CCS serves as an indicator of a molecule’s three dimensional shape in the gas phase [18–21]. IM-MS has been used by many researchers to prove that protein ions with high charge states have relatively high CCS values but less discussed is the low CCS indicative of compaction for low charge states of a protein [22–24].

Proteins in solution maintain structural integrity with noncovalent intramolecular interactions (van der Waals, hydrophobic, hydrogen bonds, salt bridges) and covalent disulfide bridges, all of which add rigidity to a molecule, limiting the conformations it can adopt. These interactions can be preserved in the gas phase [25–29] but still it is a challenging task to infer from IM-MS data alone the extent of the preservation of solution structure in the gas phase. Collapse of protein molecules to compact states in the gas phase has been reported [22] and supported by calculation [30, 31], and is a marked occurrence for intrinsically disordered proteins in solution [32, 33]. Indeed, some compaction in the structure is not unexpected as a protein undergoes desolvation from a salty solution to the solvent-free environment of a mass spectrometer; since water and counter ions are removed, free charged residues on the surface of the proteins seek to fold back and self-solvate by forming stabilizing interactions [25, 34]. Significant compaction has been reported upon transfer of proteins and protein complexes from solution to the gas phase [22, 24, 35–37], upon collisional activation [38], and this can be correlated to charge state [39]. For high charge states, as stated above, the proximity of like charges induce opposing electrostatic forces to extend the structure of the protein, and by inference narrow the available conformational space for that given charge state, this is borne out by a narrowing of the CCS distributions [15].

Recently, we have detailed how the conformational distribution of proteins in the gas phase can be altered by reducing the net charge using an approach termed ETnoD, first coined by McLuckey and co-workers [40–44]. Using electron transfer reagents in an ion mobility mass spectrometer, we have noted that under gentle activation conditions we can reduce the charge of a protein without the appearance of fragments, as in ETD [45, 46]. When electron transfer is coupled to ion mobility it is possible to monitor conformational transitions before and after electrons have been transferred to the protein. We have found that after up to two reductions in charge there is a preference for a given conformational spread, for both low and high charge states. This implies a strong correlation between charge and protein structure in the gas phase, in particular around charge states associated with partially unfolded regions of the protein. An exception to this is for very low charge states, where reduction, for example of the globular protein cytochrome *c*, causes a decrease in the CCS compared with that seen for an nESI ion of that charge state we term this compaction [47]. We have hypothesized two possible scenarios: (1) the electron pairs with a basic site (from a neutralizing contact) and removes any local Coulombic repulsion from other nearby basic groups, allowing the protein to form a more compact structure; or (2) upon electron transfer, neutralization occurs to a hydrogen bond between oppositely charged groups (salt bridge), which in turn is not as stiffly associated, again causing the protein to adopt a more compacted shape.

In this work, we extend our exploration into the role that charge plays on protein structure in the gas phase with an emphasis on this restructuring causing compaction upon reduction. As before, we reduce the charge on nESI generated protein ions and examine the CCSDs of precursor and product ions. We compare the structural change when the charge is reduced for a rigid (BPTI) [48, 49] and for an intrinsically disordered protein (beta casein) [50–52].

Intrinsically disordered proteins (IDPs), unlike structured proteins, typically exhibit a broad conformational spread in solution. When sprayed from aqueous salty solutions, IDPs have wide multimodal charge state distributions [53, 54]. Electrospray mass spectrometry coupled to ion mobility is gaining popularity as a method to study these proteins [24, 55, 56]. Thus the questions we sought to answer in this study are as followed. Given the flexible nature of disordered proteins, how stable is their structure to charge transfer processes in the gas phase? If there are variable number of basic sites available for protonation for a given structure of a given charge state, can we use ESI IM-MS to study structural change in these systems? If we reduce the charge on these structures do they show compaction to an unreduced structure of same charge state (nESI generated), is there a preference for a given conformational distribution for a given number of charge, even after multiple reductions, as we have seen before? What are the structural changes (if any) associated with a reduction in charge of a conformationally dynamic system compared with a protein known for maintaining its structural integrity in the gas phase even under harsh conditions?

## Experimental

### Samples

Bovine pancreatic trypsin inhibitor (BPTI) and beta casein were purchased from Sigma Aldrich, Irvine, UK; 1,3-dicyanobenzene was obtained from the Waters Corporation, Wilmslow, UK (Analytical Standards and Reagents); ammonium acetate was purchased from Fisher Scientific, Loughborough, UK; 1-2 mg/mL stock solutions of BPTI and beta casein were prepared in 50 mM ammonium acetate, pH 7. Prior to being used for mass spectrometry analysis, BPTI was diluted to 10 μM, whereas beta casein was desalted with biospin-6 columns (BioRad, Hecules, California, USA) then diluted before use.

### Gas Phase Charge Reduction Coupled to Ion Mobility Mass Spectrometry

Experiments were conducted on a Synapt G2 Si travelling wave ion mobility mass spectrometer (Waters Corporation, Wimslow, UK). It comprises a glow discharge source for generating radical anions [57] and a Z-spray nanoelectrospray ionization (nESI) source for generating analyte ions. Central to the instrument is the triwave region made up of three gas-filled stacked ring ion guides (SRIG) in tandem: trap, mobility cell, and transfer [58–60]. A sequential switching of polarity in the source region between negative ion (glow discharge) and positive ion (nESI) modes generate radical anions and protein cations, respectively. The protein ion of interest is selected according to mass-to-charge ratio with a quadrupole located before the trap SRIG in the instrument. Both protein cation and radical anions are transmitted to the trap SRIG post-generation in their respective sources for reaction. In each SRIG, ions are radially confined with an RF voltage and travel throughout the length of the device by application of DC voltage pulses in a stepwise manner to adjacent ring electrodes. The DC wave amplitude is optimized for charge reduction reactions in the trap SRIG where lowering of this amplitude permits radical anions to enter the trap and subsequent reduction of the protein cation. Charge-reduced product ions along with their precursor are then introduced into the adjacent gas-filled mobility cell where they undergo separation according to charge, mass, and ^TW^CCS_N2→He_. They are then transmitted to the time-of-flight (TOF) mass analyzer for measurement of their arrival times at the detector. Ion mobility data is obtained with constant travelling wave amplitude and velocity. Calibration standards are run under the same experimental conditions and collision cross-sections calculated according to a published protocol [20].

### Collision-Induced Unfolding (CIU) Coupled to Ion Mobility Mass Spectrometry

To assess the gas-phase stability of the structured BPTI, ions were *m*/*z*-selected in the quadrupole prior to injection into the trap SRIG, where the ion is collisionally activated by accelerating into neutral argon held at an approximate pressure of 2.5 × 10^–2^ mbar. Collisional activation of ions is done by controlling the kinetic energy of the ions entering the trap, which in turn is done by controlling the DC offset between the source ion guide and the trap SRIG. Arrival time distributions (ATDs) for the [M + 5H]^5+^ and [M + 6H]^6+^ ions of BPTI were generated as a function of collision energy with a collision voltage range of 2–55 V.

## Results and Discussion

### Charge Reduction of the Conformationally Restricted BPTI

**Figure 1:**
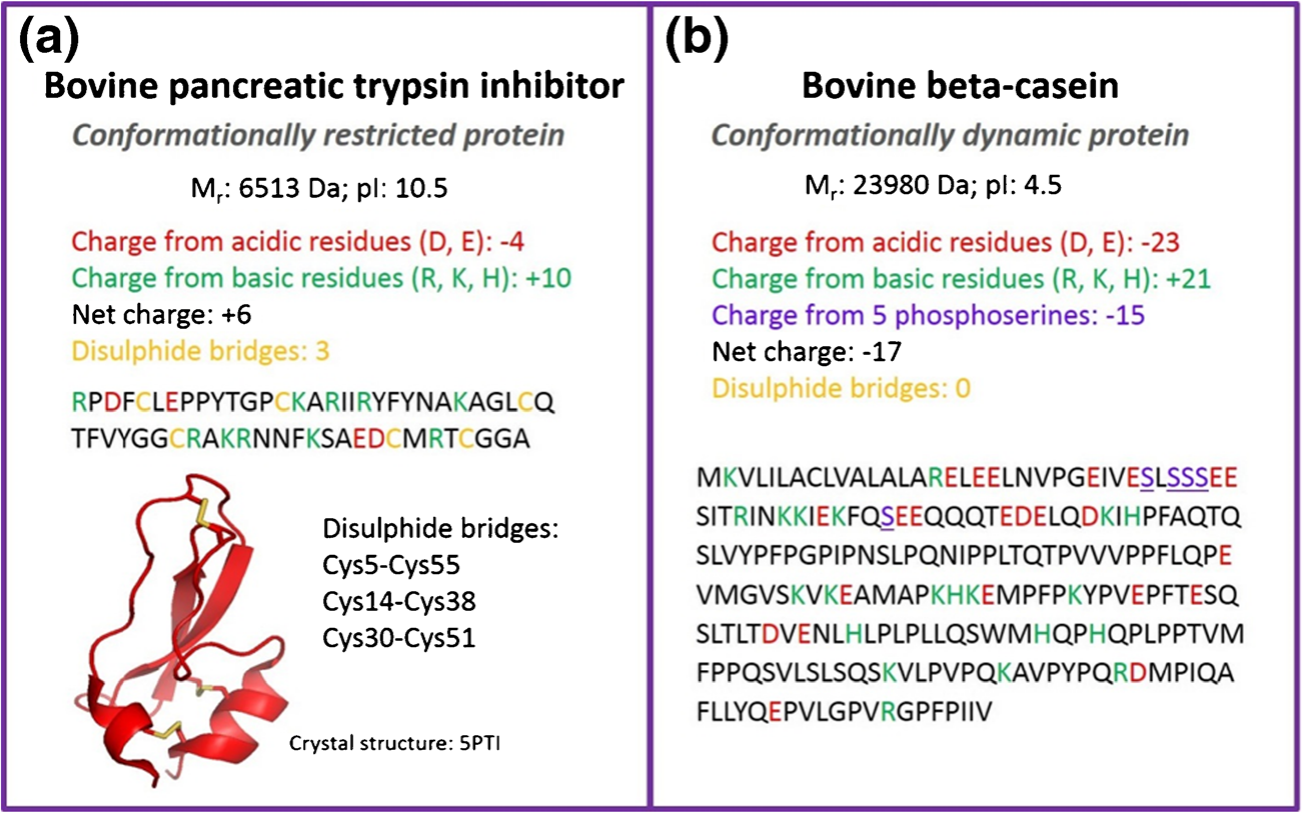
Properties of proteins used in this study: (**a**) bovine pancreatic trypsin inhibitor (BPTI), and (**b**) bovine beta-casein. BPTI serves as a model for a rigid structure whereas beta-casein represents a flexible system. Indicated are the molecular weight, isoelectric point (pI), acidic (red) and basic (green) residues, net charge, post-translational modifications, and disulphide bridges. Disulphide bridges in BPTI are shown in yellow in the crystal structure (5PTI)

**Figure 2:**
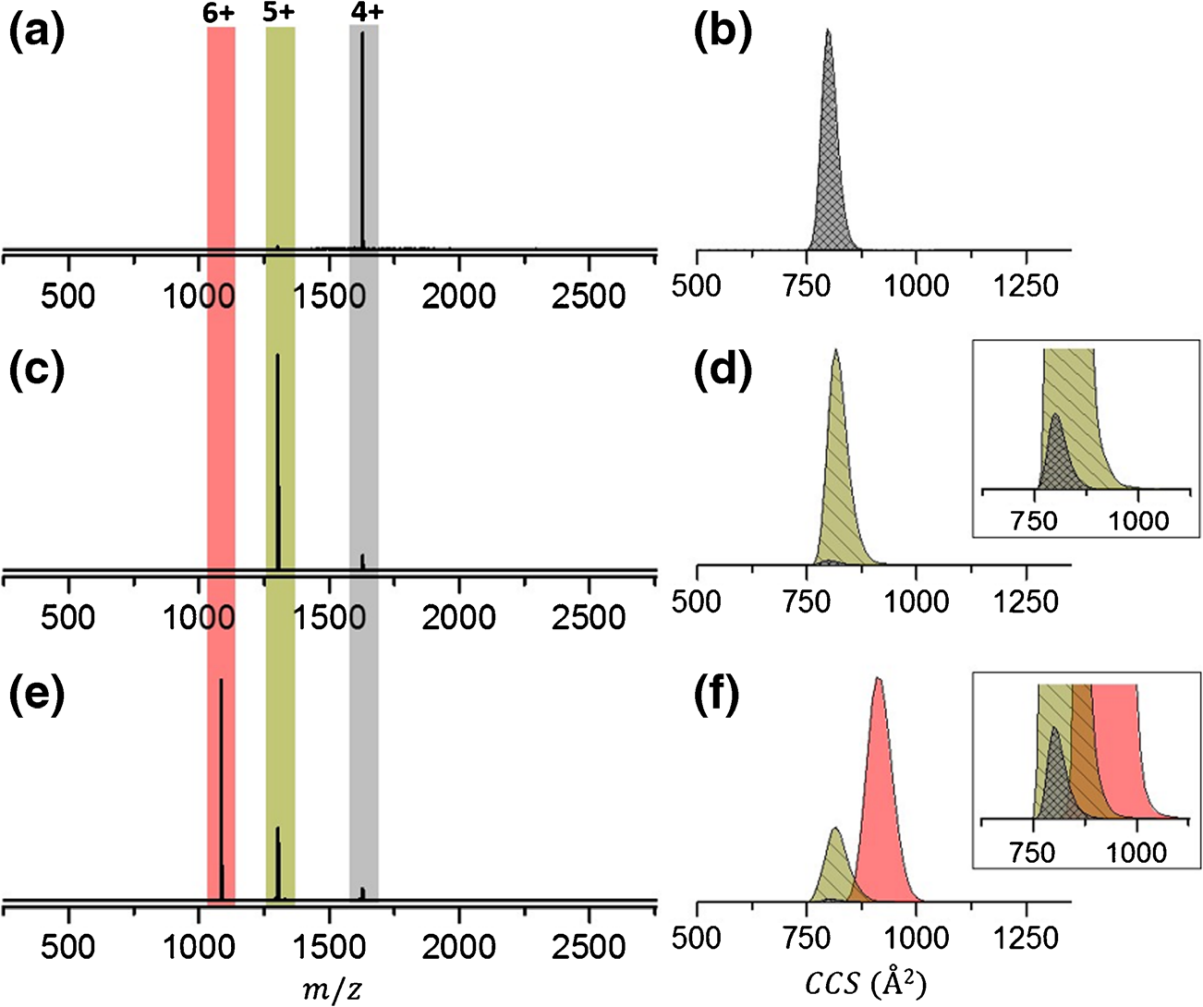
Mass spectra (**a**), (**c**), (**e**) and corresponding travelling wave collision cross-section distributions, ^TW^CCSD_N2→He_ (**b**), (**d**), (**f**) of exposed precursors and their charge-reduced products for 10 μM BPTI sprayed from a solution of 50 mM ammonium acetate, pH 7. Positively charged BPTI ions are generated via nanoelectrospray ionization and exposed to radical anions of 1,3-dicyanobenzene in the trap region of a Synapt G2 Si ion mobility mass spectrometer; *m*/*z* selected BPTI ions are reduced after which precursor and products are mobility separated and the arrival time measured from which the ^TW^CCSD_N2→He_ is determined. (**a**) Mass spectrum and corresponding (**b**) ^TW^CCSD_N2→He_ of exposed [M + 4H]^4+^ precursor of BPTI. (**c**) Mass spectrum showing the exposed [M + 5H]^5+^ precursor and its first charge-reduced product with corresponding ^TW^CCSD_N2→He_ (**d**). (**e**) Mass spectrum showing the exposed [M + 6H]^6+^ precursor and two charge-reduced products with corresponding ^TW^CCSD_N2→He_ (**f**). The inset in (**d**) and (**f**) show the expanded region from 625 to 1125 Å^2^

**Figure 3:**
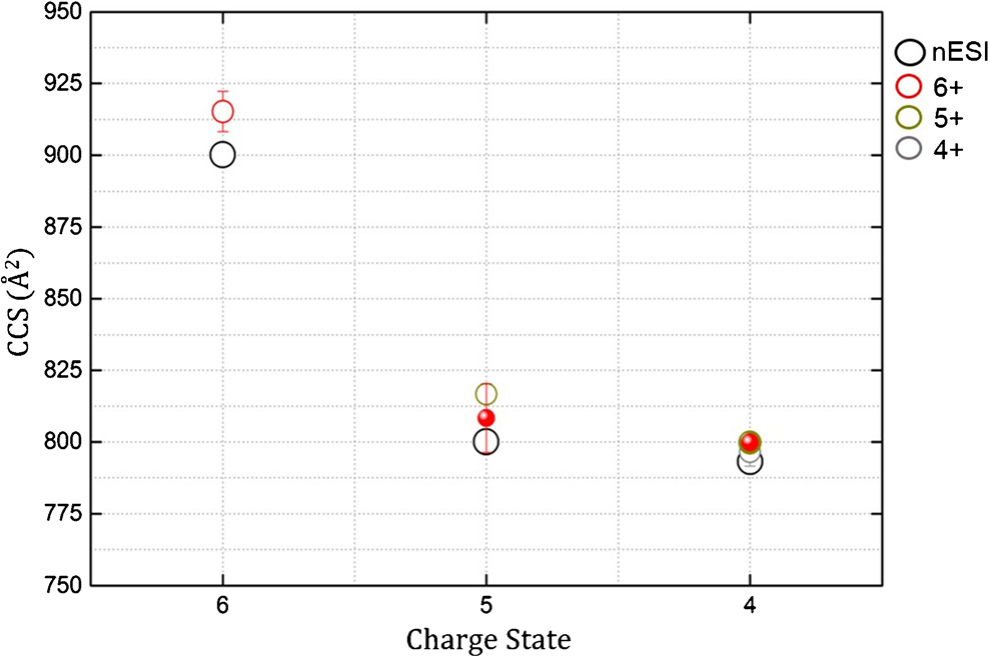
Plot showing the charge state versus the collision cross-section (^TW^CCS_N2→He_) of BPTI where ions are generated via nanoESI ionization and sprayed from 50 mM ammonium acetate, pH 7. The unexposed precursors, nanoESI generated ions, [M + 6H]^6+^, [M + 5H]^5+^, and [M + 4H]^4+^, are represented by open black circles. Ions exposed to radical anions are represented by open circles of different colors: red - [M + 6H]^6+^, dark yellow - [M + 5H]^5+^, grey - [M + 4H]^4+^. Charge-reduced products are shown in the same color as the precursor from which they originate but represented by solid dots

The nESI mass spectrum of BPTI sprayed from 50 mM ammonium acetate, pH 7, displays a narrow CSD with three species detected, [M + 4H]^4+^, [M + 5H]^5+^, and [M + 6H]^6+^ (Supplementary Figure S1). This is expected as BPTI is a 6.5 kDa monomeric protein, the compact three-dimensional structure of which is held in place with three intramolecular disulfide bridges (Figure 1) [48, 49]. A previous report indicates that BPTI retains a compact structure in the gas phase even when it has undergone in-source collisional activation [25]. The same study reports gas-phase collision cross-sections of ESI generated ions measured in helium buffer gas, ^TW^CCS_He_, for each charge state as 770 Å^2^ (4+), 790 Å^2^ (5+), 860 Å^2^ (6+), and 960 Å^2^ (7+), and the crystal structure, 5PTI [61] gave a theoretical CCS_He_, via the projection approximation method [62, 63] of 767 Å^2^. Our measurements done on a travelling wave instrument in nitrogen buffer gas and converted to helium cross-sections, ^TW^CCS_N2→He_, using the latter measurements, are centered at 793 Å^2^ (4+), 800 Å^2^ (5+), and 900 Å^2^ (6+), each charge state showing a single sharp peak in the collision cross-section distribution (^TW^CCSD_N2→He_). The narrow ^TW^CCSD_N2→He_ indicate that each charge state of BPTI is present as a compact conformer in the gas phase. The trap SRIG on a Synapt travelling wave ion mobility instrument can be used as a reaction chamber where charge reduction of an isolated nESI generated protein species can occur by exposure to radical anions. Thus, to investigate the conformational change when each charge state of BPTI is *m*/*z*-selected and subjected to charge reduction in the trap SRIG of the instrument by exposure to radical anions of 1,3-dicyanobenzene, we compare the CCSD of precursors and products before and after reduction (Figure 2). We see no evidence of charge reduction for the lowest charge state precursor, [M + 4H]^4+^, upon exposure to radical anions of 1,3-dicyanobenzene (Figure 2a). The ^TW^CCSD_N2→He_ is a single peak indicative of a compact conformer centered at 796 Å^2^. The [M + 5H]^5+^ precursor (817 Å^2^) undergoes a single reduction to form the [M + 5H]^4+·^ with a ^TW^CCSD_N2→He_ centered at 800 Å^2^ (Figure 2c, d), whereas the [M + 6H]^6+^ precursor (915 ± 7 Å^2^) undergoes two reductions to the [M + 6H]^5+·^ and [M + 6H]^4+··^ products (Figure 2e, f) with ^TW^CCSD_N2→He_ centered at 808 ± 12 Å^2^ and 800 Å^2^, respectively. In all cases, regardless of the charge state of precursor, reduction has occurred to a product with four charges with similar ^TW^CCSD_N2→He_. The transition of 6+ to 4+ shows the intermediate 5+ as having a slightly larger ^TW^CCS_N2→He_, but within standard error, than that of the exposed 5+ ion (Figure 3), whereas the 4+ ion in the presence of radicals and originating from the charge reduction of higher charge states (6+ and 5+) shows identical ^TW^CCS_N2→He_ within error. All ^TW^CCS_N2→He_ values are shown in Supplementary Table S1. The lack of further compaction is evidence against reduction of any of the three disulfide bonds present in BPTI, since this would likely decrease the ^TW^CCS_N2→He_ of the protein ion allowing tighter packing (for low charge states at least). This is somewhat surprising, since disulfide bond cleavage upon exposure to electrons and radical anions in the gas phase is well reported [64–66].

The isotopic distribution of the nESI-generated ions and product ions up to two reductions is shown in Supplementary Figure S2. The molecular weight measured does not indicate that the disulfides have been reduced during ESI. Supplementary Figure S2a shows the isotopic distribution generated for the [M + 4H]^4+^ ion. This fits to a theoretical distribution shown in solid black dots. The first charge-reduced product originating from the [M + 5H]^5+^ precursor fits a theoretical isotopic distribution for the ETnoD product, [M + 5H]^4+^ (Supplementary Figure S2b, open circles). Similarly, the second charge-reduced product originating from the [M+6H]^6+^ precursor matches well to the theoretical isotopic distribution for the ETnoD product, [M + 6H]^5+^ (Supplementary Figure S2c, open circles). This indicates that the predominant reaction responsible for charge reduction is electron transfer from the radical anion to the protonated precursor.

### Gas-Phase Stability of BPTI in the Trap SRIG of a Travelling Wave Instrument Assessed by CIU

**Figure 4:**
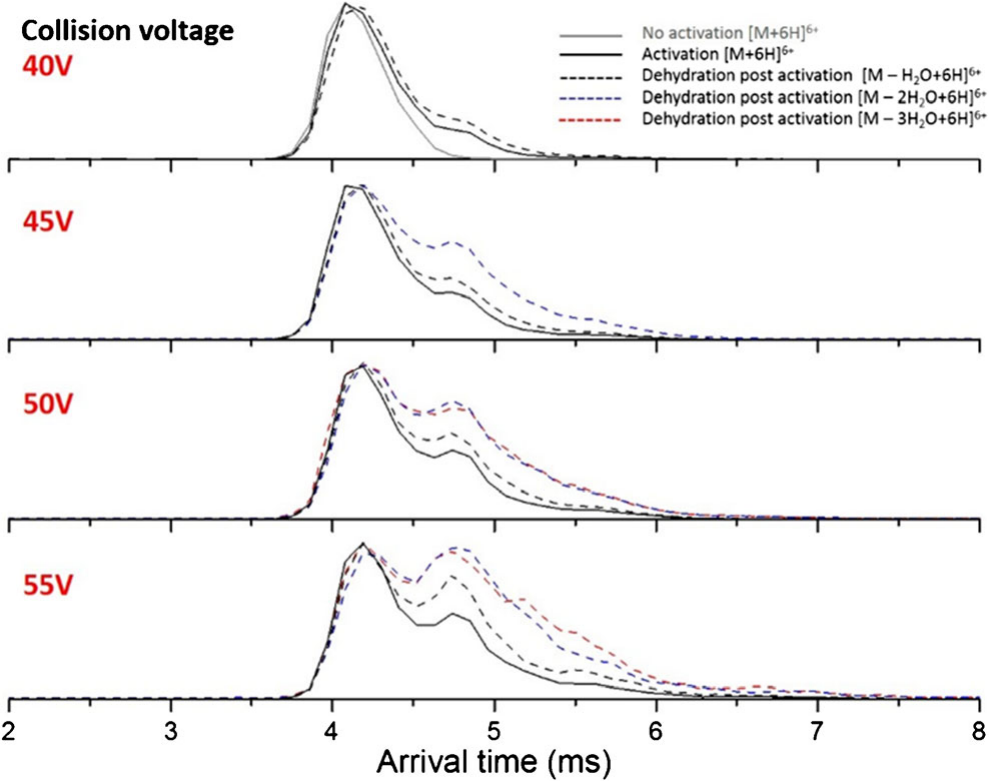
Collision induced unfolding (CIU) of the [M + 6H]^6+^ ion of BPTI. Arrival time distributions (ATDs) for the nonactivated and activated plus dehydrated forms of of BPTI are shown. Upon collisional activation with collision voltage 40–55 V, an enhancement in the abundance of the more extended structure is seen with further enhancement upon dehydration, in constrast to ETnoD experiments where the compact conformer is retained after and up to two reductions

We next set about probing the stability of this compact BPTI conformer using collision-induced unfolding. In previous work, Shelimov et al. performed collisional heating of BPTI by raising the injection energy in the source region of an IM-MS instrument before ion mobility measurements, and reported no significant change in the drift time distributions [25]. Since the instrumental conditions setup under which the latter work was undertaken are different from this study, it is of interest to us to assess the stability of BPTI in the gas phase under our instrumental conditions. The gas-phase stability of BPTI is assessed by collision-induced unfolding experiments. Supplementary Figure S3a and b show the results of these experiments for the [M + 5H]^5+^ and [M + 6H]^6+^ ions of BPTI. A single peak for both charge states is observed as the collision voltage is increased up until 30 V, indicative of the retention of a compact conformer. At 35 V (lab frame CE) there is a broadening of the ATD and the appearance of another species with a later arrival time indicative of an unfolded form. [M + 6H]^6+^ is activated to a single unfolded form at the maximum collision voltage (55 V) used in these experiments (Supplementary Figure S3a-left), whereas the [M + 5H]^5+^ species show the appearance of three unfolded forms with same (lab frame) energy input (Supplementary Figure S3b-left). The corresponding mass spectra for each ATD at each collision voltage is shown to the right of Supplementary Figure S3. For both charge states, above a collision voltage of 35 V fragment ions appear corresponding to dehydrated forms of each precursor. The extracted ATDs for the activated dehydrated peaks of the [M + 6H]^6+^ precursor are shown in Figure 4. The collisionally activated forms are shown for collision voltages 40–55 V as the appearance of an extended form is distinct from 40 V (Supplementary Figure S3b – left) and the appearance of a dehydrated form of the precursor is also clearly evident in the corresponding mass spectrum (Supplementary Figure S3b – right). In Figure 4, the ATD of the nESI generated form (no activation) showing the single compact conformer is shown in grey. The collisionally activated forms for collision voltages 40–55 V are shown in solid black lines, as the energy increases the arrival time of the protein increases. More of the extended form is seen for the dehydrated species (extracted ATDs for the loss of one, two, and three water molecules are seen in dashed lines colored black, blue, and red, respectively). Dehydration of peptides and proteins is a common occurrence in the gas phase of a mass spectrometer, in most cases upon collisional activation [67]. This work shows that dehydration also destabilizes the protein with respect to the intact form indicating that the water has been lost from a stabilizing interaction, likely between an acidic group and a proton.

### Charge Reduction of the Intrinsically Disordered Beta-Casein

The nESI mass spectrum of beta casein sprayed from 50 mM ammonium acetate, pH 7, shows a multimodal distribution of charge states with 9 ≤ *z* ≤ 28 for [M + zH]^z+^. (Supplementary Figure S4) as reported previously [11]. This comprises conformational families with ^TW^CCSD_N2→He_ 2991 ± 82 Å^2^ for the [M + 12H]^12+^ to 5638 ± 113 Å^2^ for the [M + 25H]^25+^ (Supplementary Figure S5). This wide range is correlated to many dynamic unstructured forms of beta casein in solution [68]. In solution, beta casein has a high net charge (–13 at pH 6.6) [69] and is amphipathic, which is attributed to the uneven distribution of hydrophobic and hydrophilic residues with a high charge density on the N-terminus. (Figure 1). Its charged side chains are 5 histidines and 5 phosphoserines, all which are deprotonated in solution at pH 7 [70]. It has an isoelectric point of 4.5 and a net protonated charge of –22 in aqueous solutions of sodium chloride in the pH range 5.5–10.5 [71]. The high number of charged groups, coupled with the lack of tertiary structure, is indicative of the possibility of a wide conformational spread.

The ^TW^CCSD_N2→He_ from charge states 12 ≤ *z* ≤ 25 reveal a sharp transition from the compact unimodal form for [M + 12H]^12+^, to multiple conformers for 13 ≤ *z* ≤ 18, reverting to unimodal forms at higher charge states, 19 ≤ *z* ≤ 25 presenting more narrow ^TW^CCSD_N2→He_ (Supplementary Figure S5). De la Mora derived an empirical relationship, which gives the upper limit of charge that a globular protein can hold and is oftentimes used to indicate the boundary at which a protein transitions from a globular (ideally spherical) structure to partially folded structures [72]. This relationship is given by:

$$ {Z}_R=0.0778\sqrt{m} $$

where ${Z}_R$ is the upper limit of charge and *m* is the protein mass. For beta casein, ${Z}_R$ equals 12. This correlates with our measured ^TW^CCSD_N2→He_ in the transition from a compact conformer to partially folded states with multiple conformers – the order to disorder transition (Supplementary Figures S4 and S5).

**Figure 5:**
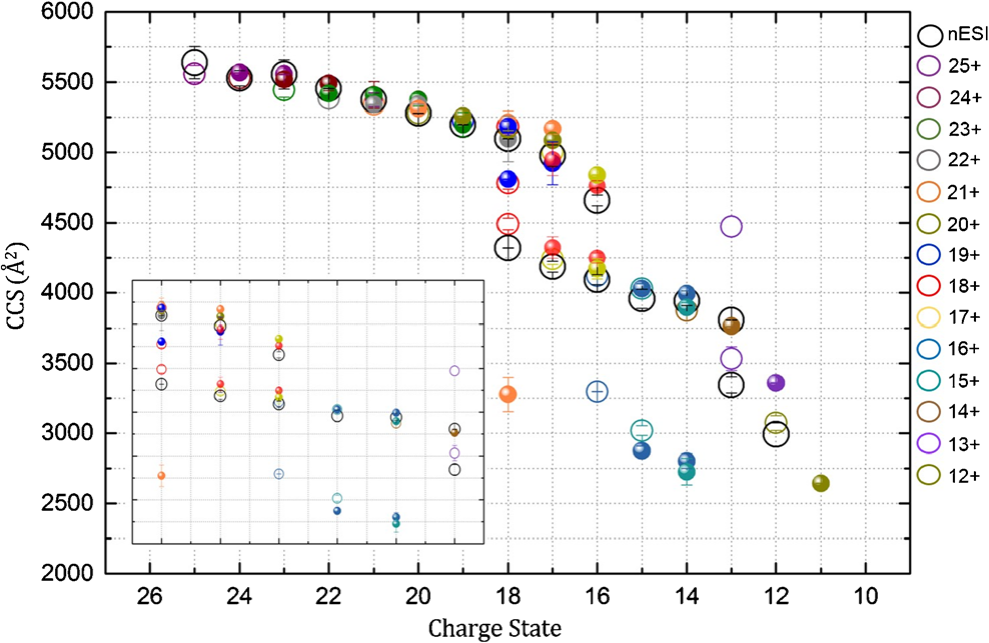
Plot showing the charge state versus collision cross-section (^TW^CCSD_N2→He_) of beta casein sprayed from 50 mM ammonium acetate, pH 7 for ions generated via nanoESI ionization (precursors unexposed to ETD reagent), ions exposed to the ETD reagent (radical anions of 1,3-dicyanobenzene) in the trap SRIG of a travelling wave ion mobility mass spectrometer, and their charge-reduced products. The unexposed precursors, with charge states ranging from 12+ to 25+ are represented by open black circles. Exposed precursors are represented by open circles of different colors, whereas their corresponding products are displayed in solid dots of same color. Compaction and structural rearrangement depends upon different charge state regimes: low, intermediate, and high. The inset shows an expanded view for intermediate charge states

We investigate here the effect of charge on the structure of beta casein as we reduce the net charge via electron transfer from radical anions. In order to best report on the conformational change, each charge state of beta casein is individually *m*/*z*-selected and exposed to radical anions in the trap SRIG of the instrument. We divide the charge state distribution into three regions, low (9+ to 12+), intermediate (13+ to 18+), and high (19+ to 28+), and compare the conformational change in each region as the charge is systematically reduced on individual ions. Figure 5 shows the relationship between charge state and collision cross-section for beta casein cations (reduced and unreduced). The nESI generated precursors, unexposed to radical anions, are shown in black open circles. The corresponding ^TW^CCSD_N2→He_ previously mentioned are shown in Supplementary Table S2. Ions exposed to radical anions are shown in open circles whereas their charge-reduced products are shown in solid colored dots of the same color as the precursor. For example, the exposed 23+ ion and its four charge-reduced products are represented as an open green circle and solid green dots, respectively. We cannot confirm whether charge reduction is predominantly attributable to electron transfer from radical anion to protein or proton transfer from the protein to the radical anion, as we are unable to detect both precursor and product ions with isotopic resolution. Instead, we observed broad mass spectral peaks with many salt adducts regardless of charge state. This is a common occurrence for some IDPs as they possess a greater number of charged residues giving a higher tendency for adduct formation by charged groups paring with available salts in comparison with a structured protein. In addition, the heterogeneity of beta casein due to the presence of phosphorylated residues (Figure 1) will also contribute to broad mass spectral peaks.

### Comparison of the Collision Cross-Section Distributions (^TW^ CCSD_N2__→__He_) for Specific Charge States of the Disordered Beta Casein

#### Low and High Charge States

**Figure 6:**
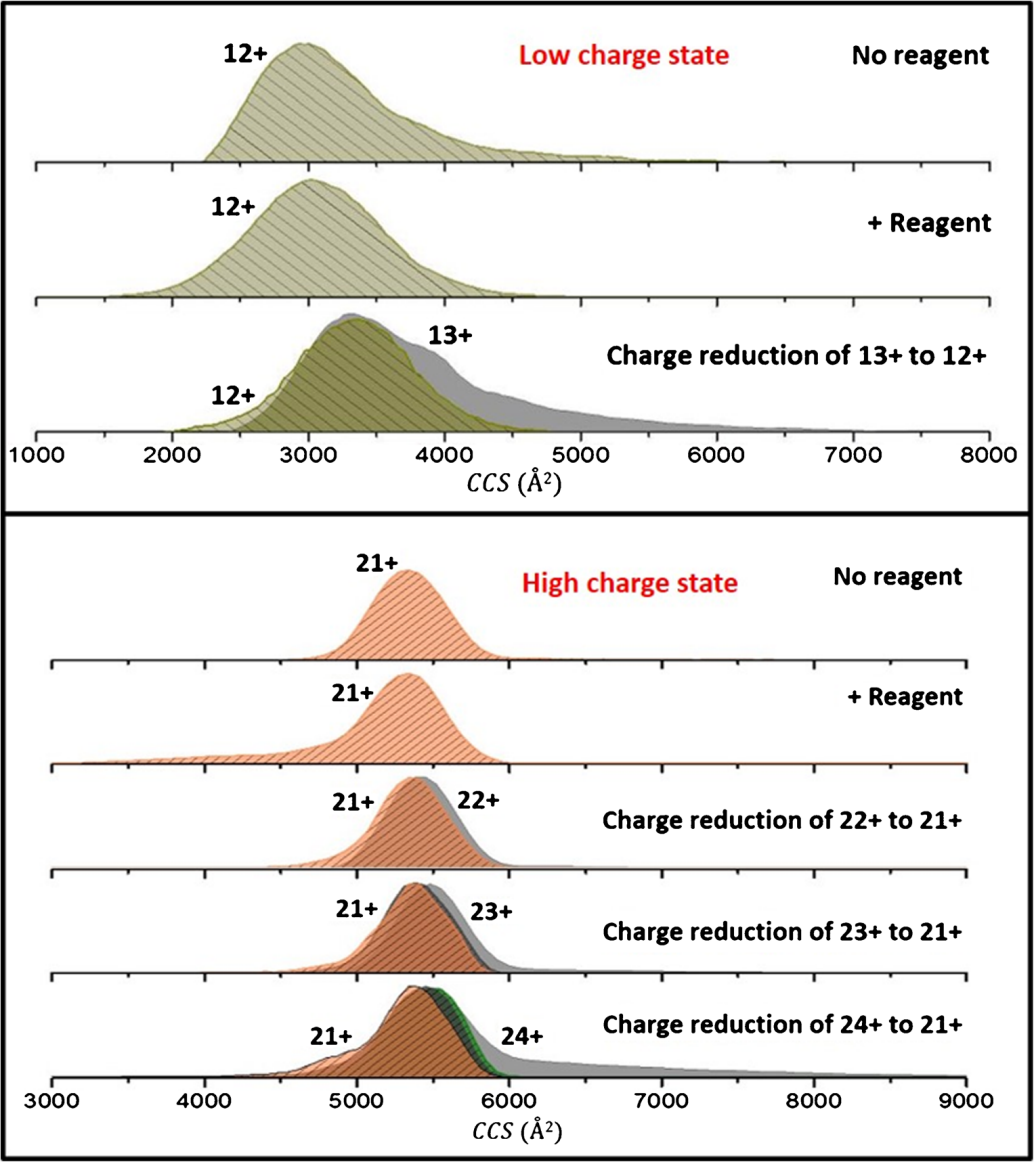
Collision cross-section distributions (^TW^CCSD_N2→He_) of the [M + 12H]^12+^ and [M+ 21 H]^21+^ ions (representative of low and high charge states) of beta casein, a disordered protein. Ions were generated from electrospraying out of ammonium acetate, pH 7. The ^TW^CCSD_N2→He_ shown represent unexposed precursors (No reagent), the result of exposure of these same ions to radical anions (+Reagent), and their products of the same charge originating from different precursors and obtained by charge reduction

In the case of lower charge states, we see some compaction *cf*. of the nESI form in the presence of radical anions. As an example, the 12+ ion before and after exposure to radical anions is compared along with its charge-reduced form originating from the 13+ (Figure 6a). There is a slight decrease in the width of the ^TW^CCSD_N2→He_ of the 12+ upon exposure to radicals; however, the mean ^TW^CCSD_N2→He_ is within error of that reported for the nESI generated ion. We cannot compare the reduced 12+ form originating from any other precursor as there is (interestingly) no reduction to the 12+ ion from any higher charged precursor ion (Figure 5). The nESI-generated 12+ ion has a ^TW^CCSD_N2→He_ centered at 2991 ± 82 Å^2^, whereas that of the exposed form is centered at 3072 ± 53 Å^2^. By comparison, the 12+ reduced form originating from the 13+ precursor (3345 ± 59 Å^2^ and 3804 ± 1 Å^2^) has a ^TW^CCSD_N2→He_ centered at 3358 ± 2 Å^2^. Of note is the ^TW^CCSD_N2→He_ of the 13+ precursor with two overlapping conformers, which are converted to one on single reduction to the 12+ ion; noteworthy is that the reduced 12+ has a larger ^TW^CCSD_N2→He_ than the nESI generated 12+. Overall, the lower charge states, (12+ and 11+) undergo only up to two reductions compared with higher charge states where we are able to see up to four reductions under the same experimental conditions (Figure 5).

The degree of structural rearrangement in unfolded forms of beta casein (higher charge states) is small in magnitude relative to the number of charge reductions seen (Figures 5 and 6b). For example, the 24+ charge state, which in the presence of radical anions (shown by a solid wine colored dot in Figure 5) transitions from a distribution with a ^TW^CCSD_N2→He_ centered at 5528 ± 54 Å^2^ to 5376 ± 45 Å^2^ after four reductions. The 21+ transitions from a ^TW^CCSD_N2→He_ centered at 5341 ± 1 Å^2^ to 5168 ± 39 Å^2^ also after four reductions. An examination of the ^TW^CCSD_N2→He_ for the 21+ originating from three different precursors (22+, 23+, and 24+) is shown in Figure 6b. The 21+ precursor unexposed and exposed to radical anions have ^TW^CCSD_N2→He_ centered at 5375 ± 48 Å^2^ and 5341 ± 1 Å^2^, respectively. The charge-reduced products with a charge of 21+, originating from the 22+ (5455 ± 1 Å^2^), 23+ (5554 ± 102 Å^2^), and 24+ (5528 ± 55 Å^2^), have ^TW^CCSD_N2→He_ centered at 5341 ± 1 Å^2^, 5409 ± 1 Å^2^, and 5409 ± 95 Å^2^, respectively. The distributions for 24+, 23+, and 22+ precursors slightly shift to lower ^TW^CCS_N2→He_ upon reduction to the 21+. The same is true for other higher charge states representing extended structures (Figure 5). Although we see a decrease in the ^TW^CCSD_N2→He_ for higher charge states, it is not as dramatic a change (compaction and structural rearrangement) as that reported for intermediate charge states, *vide infra*, indicative that there are not enough noncovalent interactions in the unfolded forms to facilitate any collisional compaction and that Coulombic repulsion still dominates the conformations (Figure 5).

#### Intermediate Charge States

**Figure 7:**
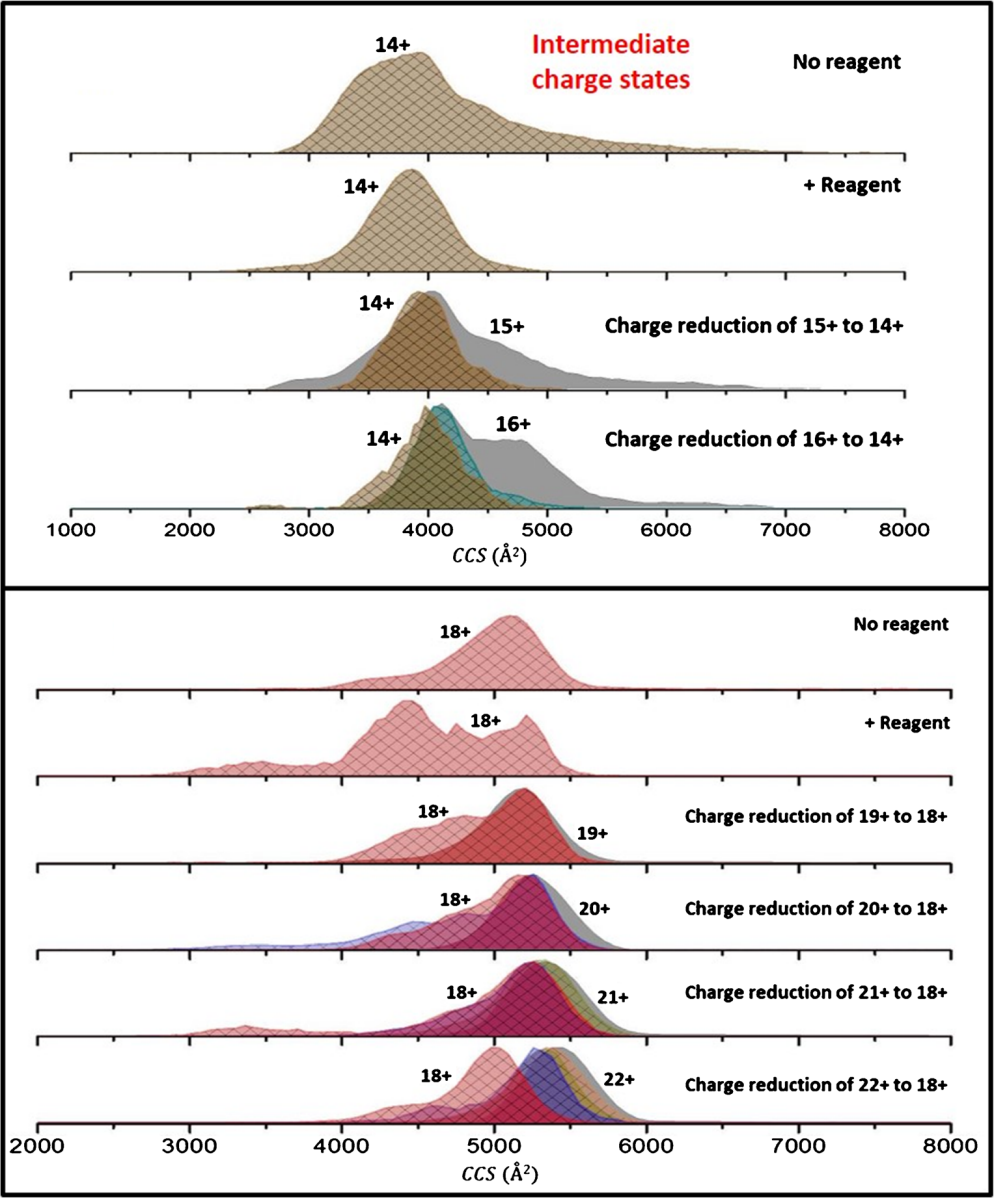
Collision cross-section distributions (^TW^CCSD_N2→He_) of the [M + 14H]^14+^ and [M + 18H]^18+^ of beta casein generated from electrospraying out of ammonium acetate, pH 7. The ^TW^CCSD_N2→He_ shown represent unexposed precursors  (No reagent), the result of exposure of these same ions to radical anions (+Reagent), and ions of the same charge originating from different precursors and obtained by charge reduction

Intermediate charge states show the most interesting behavior compared with the low and high charge states. We see both reduction in ^TW^CCS_N2→He_ and rearrangement (but no charge-reduced product with a larger ^TW^CCS_N2→He_ than its precursor); we attribute this to the conformational flexibility of these states, which appear as an intermediate between the Coulombic forces that dictate the structures of these ions in the gas phase, as indicated by the presence of multiple conformers (Supplementary Figure S5). We exemplify this with the ^TW^CCSD_N2→He_ of the 14+ and 18+ precursors and the reduced form of these ions originating from higher charge states (Figure 7). The nESI-generated 14+ ion (Figure 7a) show a broad conformational spread with multiple conformers that are poorly resolved and peaks at 3940±32 Å^2^. Upon exposure to radical anions, we see a narrowing of the ^TW^CCSD_N2→He_ to a single peak with a ^TW^CCSD_N2→He_ centered at 3873 ± 1 Å^2^. We have observed this before and attributed this to collisional cooling and ion–ion interactions [47]. The nESI 15+ shows a broad distribution with multiple conformers that are poorly resolved, with a main peak at 3957 ± 68 Å^2^. The charge-reduced 14+ originating from the 15+ precursor shows a more narrow distribution than the nESI 14+ with a peak centered at 3895 ± 32 Å^2^ and a shoulder to its right. Similarly, the charge-reduced 14+ originating from the 16+ precursor (^TW^CCS_N2→He_ peaks at 4093 ± 36 Å^2^ and 4658 ± 36 Å^2^) peaks at 3993 ± 21 Å^2^ with a shoulder to its right. Once again the reduced 14+ shows a more narrow distribution than the nESI 14+.

Our second intermediate charge state example is the 18+ ion and the reduced forms originating from higher charge state precursors (Figure 7b). The nESI generated ion for the 18+ charge state of beta casein shows two conformers with ^TW^CCSD_N2→He_ centered at 4230 ± 1 Å^2^ and 5097 ± 1 Å^2^. Upon exposure to radical anions, we see significant compaction and rearrangement with a broad ^TW^CCSD_N2→He_ and multiple conformers. The 19+, 20+, 21+, and 22+ precursors (grey in each of the bottom four traces in Figure 7b) all present as single conformers with ^TW^CCSD_N2→He_ centered at 5198 ± 1 Å^2^, 5279 ± 1 Å^2^, 5375 ± 48 Å^2^, and 5455 ± 1 Å^2^. The ^TW^CCS_N2→He_ of each of these precursors decreases as they reduce to the 18+ form (Figures 5 and 7b). The ^TW^CCSD_N2→He_ of the reduced 18+ in each instance is broad and multimodal. When the 21+ ion (^TW^CCS_N2→He_ 5375 ± 48 Å^2^) undergoes three reductions, it is significantly altered to a ^TW^CCSD_N2→He_ with maxima at 3276 ± 123 Å^2^, 5213 ± 18 Å^2^ (Figure 7b).

The conformational rearrangement of the intermediate charge states of beta casein upon reduction indicates that there are significant numbers of intramolecular interactions that can alter as the net charge is reduced, allowing the protein to readily access new conformations. This contrasts with the behavior of cytochrome *c* and myoglobin, sprayed from a denaturing solvent, reported in our previous publication [47], and with the rigid BPTI herein (Figures 2 and 3), indicating the plasticity of the conformational landscape for this IDP. Further, this suggests that salt bridge rearrangement or new salt bridges could occur during the desolvation process, post-activation and post-charge reduction, a phenomenon recently suggested by Loo et al. and Zhang and Vachet [73, 74].

#### Charge Reduction – Structured Versus Disordered

This study is an extension of our previous work where we examined the change in conformation upon charge reduction of two globular proteins, cytochrome *c* and myoglobin, in both aqueous salt and denaturing conditions [47]. Our major findings in the latter study was conformer-selective reactivity; a depletion of the compact conformer for all charge states was seen when exposed to radical anions. There is precedent for this in the literature where a depletion in the abundance of compact and partially folded conformers of protonated ubiquitin ions occurred upon charge stripping when exposed to bases in the gas phase [75]. Our observations were the same regardless of charge state, a single exception being for a low charge state of cytochrome *c*, sprayed from aqueous salt, where compaction in the radical product [M + 7H]^6+•^ formed from the [M + 7H]^7+^ is observed. This is the basis of the current study where we sought to explore compaction upon charge reduction in relation to conformational flexibility. Overall reduction in charge of BPTI results in a reduction in the ^TW^CCS_N2→He_ to single conformers that resemble unreduced forms of the same charge (Figures 2 and 3) as we saw for cytochrome *c* and myoglobin under native conditions. We infer that upon electron transfer reduction relieves local repulsion (6+ → 5+ → 4+) to produce a final structure resembling that of the nESI ion of same charge. Interestingly, when we collisionally activate BPTI, dehydrated forms of the gas-phase molecule extend more readily, suggesting that the water loss has disrupted a stabilizing contact (Figure 4 and Supplementary Figure S3).

The behavior of the unstructured beta casein upon charge reduction is not as clear-cut. Upon systematic reduction of the charge state, we probe the role that charge–charge interactions play in maintaining conformational families of unstructured proteins (Figures 5, 6, and 7). The highest number of reductions (4) occur with the highest charge states but these are not accompanied by any significant change in conformation. This indicates that Coulombic repulsion still dominates the gas-phase conformations and that the noncovalent interactions are too few to enable any collision-induced coalescence of the protein on removal of one or more charged site.

Lower charge states show at most two reductions in charge and some slight reduction in ^TW^CCS_N2→He_ (Figure 6). The greatest effect is for intermediate charge states where collapse to compact states as well as structural rearrangement is seen. For these intermediate charge state structures, there are multiple ways in which a structure can be arranged once a neutralizing contact is disrupted via charge reduction. We propose that this is indicative of substantial restructuring of this flexible unstructured molecule, imparted by a change in the electrostatics along with other noncovalent interactions.

## Conclusions

In this study, we monitor the change in gas-phase structure of the rigid BPTI and the conformationally dynamic beta casein as a function of charge where different charge states are representative of different degrees of compactness or disorder. We report the change in conformation as each charge state of each protein undergoes reduction upon exposure to radical anions of 1,3-dicyanobenzene. For the conformationally restricted BPTI, regardless of the precursor ion, reduction results in a smaller unimodal ^TW^CCSD_N2→He_. An indication of the influence of charged group interactions is provided by our CIU data where the dehydrated forms of BPTI unfold more readily, which may be explained by water loss from an acidic side chain (or the C terminus) interacting with a proton.

In contrast, the intrinsically disordered protein beta casein shows charge state-dependent conformational change upon exposure to radical anions and reduction in charge. Partially folded conformers (intermediate charge states) of beta casein exhibit high conformational flexibility upon reduction in charge, whereby reduction of the mean ^TW^CCS_N2→He_ is accompanied by significant conformational rearrangement. More extended structures (higher charge states) show compaction upon charge reduction to ^TW^CCS_N2→He_ comparable to those measured from ions generated from nESI, whereas low charge states reduce in ^TW^CCS_N2→He_ but sometimes with ^TW^CCS_N2→He_ larger than those measured for the nESI forms and with the formation of multiple conformers.

The ease with which the conformation of both proteins can alter upon reaction strikes a cautionary note in that such processes readily occur in electrospray. Overall, this study illustrates the ease with which conformations can be manipulated for beta casein, an intrinsically dynamic protein, by altering its net charge, and most interestingly that a number of very different conformations can be found for a given charge state for beta casein, versus a much narrower landscape for the structured BPTI.

Future studies will demonstrate whether these effects are inherent to intrinsically disordered proteins or whether the protein’s sequence is the primary cause for such effects. Investigations will include effects of protein size, isoelectric point (pI), post-translational modifications and charged groups, solution conditions that affect structural stability, and the effect of different charge-reducing reagents (both proton and electron transfer reagents) on conformation.

## Electronic supplementary material

Below is the link to the electronic supplementary material.

ESM 1 (DOCX 633 kb)

## References

[1] Chowdhury SK, Katta V, Chait BT (1990). Probing conformational changes in proteins by mass-spectrometry. J. Am. Chem. Soc..

[2] Katta V, Chait BT (1991). Observation of the heme-globin complex in native myoglobin by electrospray-ionization mass spectrometry. J. Am. Chem. Soc..

[3] Mao D, Babu KR, Chen YL, Douglas DJ (2003). Conformations of gas-phase lysozyme ions produced from two different solution conformations. Anal. Chem..

[4] Wang G, Cole RB (1994). Effect of solution ionic strength on analyte charge state distributions in positive and negative ion electrospray mass spectrometry. Anal. Chem..

[5] Mirza UA, Chait BT (1994). Effects of anions on the positive ion electrospray ionization mass spectra of peptides and proteins. Anal. Chem..

[6] Konermann L, Douglas DJ (1997). Acid-induced unfolding of cytochrome *c* at different methanol concentrations: electrospray ionization mass spectrometry specifically monitors changes in the tertiary structure. Biochemistry.

[7] Katta V, Chait BT (1991). Conformational changes in proteins probed by hydrogen-exchange electrospray-ionization mass spectrometry. Rapid Commun. Mass Spectrom..

[8] Ehrmann BM, Henriksen T, Cech NB (2008). Relative importance of basicity in the gas phase and in solution for determining selectivity in electrospray ionization mass spectrometry. J. Am. Soc. Mass Spectrom..

[9] Schnier PD, Gross DS, Williams ER (1995). Electrostatic forces and dielectric polarizability of multiply protonated gas-phase cytochrome c ions probed by ion/molecule chemistry. J. Am. Chem. Soc..

[10] Beveridge R, Phillips AS, Denbigh L, Saleem HM, MacPhee CE, Barran PE (2015). Relating gas phase to solution conformations: lessons from disordered proteins. Proteomics.

[11] Beveridge R, Covill S, Pacholarz KJ, Kalapothakis JMD, Macphee CE, Barran PE (2014). A mass-spectrometry-based framework to define the extent of disorder in proteins. Anal. Chem..

[12] Konermann L, Douglas DJ (1998). Equilibrium unfolding of proteins monitored by electrospray ionization mass spectrometry: distinguishing two-state from multi-state transitions. Rapid Commun. Mass Spectrom..

[13] Schnier PD, Gross DS, Williams ER (1995). On the maximum charge state and proton transfer reactivity of peptide and protein ions formed by electrospray ionization. J. Am. Soc. Mass Spectrom..

[14] Williams ER (1996). Proton transfer reactivity of large multiply charged ions. J. Mass Spectrom..

[15] Clemmer DE, Jarrold MF (1997). Ion mobility measurements and their applications to clusters and biomolecules. J. Mass Spectrom..

[16] Lanucara F, Holman SW, Gray CJ, Eyers CE (2014). The power of ion mobility-mass spectrometry for structural characterization and the study of conformational dynamics. Nat. Chem..

[17] St. Louis RH, Hill HH, Eiceman GA (1990). Ion mobility spectrometry in analytical chemistry. Crit. Rev. Anal. Chem..

[18] Faull PA, Florance HV, Schmidt CQ, Tomczyk N, Barlow PN, Hupp TR, Nikolova PV, Barran PE (2010). Utilizing ion mobility-mass spectrometry to interrogate macromolecules: Factor H complement control protein modules 10–15 and 19–20, and the DNA-binding core domain of tumor suppressor p53. Int. J. Mass Spectrom..

[19] Jurneczko E, Barran PE (2011). How useful is ion mobility mass spectrometry for structural biology? The relationship between protein crystal structures and their collision cross-sections in the gas phase. Analyst.

[20] Ruotolo BT, Benesch JLP, Sandercock AM, Hyung S-J, Robinson CV (2008). Ion mobility-mass spectrometry analysis of large protein complexes. Nat. Protoc..

[21] Bohrer BC, Merenbloom SI, Koeniger SL, Hilderbrand AE, Clemmer DE (2008). Biomolecule analysis by ion mobility spectrometry. Annu. Rev. Anal. Chem..

[22] Pagel K, Natan E, Hall Z, Fersht AR, Robinson CV (2013). Intrinsically disordered p53 and its complexes populate compact conformations in the gas phase. Angew. Chem. Int. Ed..

[23] Laszlo KJ, Munger EB, Bush MF (2016). Folding of protein ions in the gas phase after cation-to-anion proton-transfer reactions. J. Am. Chem. Soc..

[24] Jurneczko E, Cruickshank F, Porrini M, Clarke DJ, Campuzano IDG, Morris M, Nikolova PV, Barran PE (2013). Probing the conformational diversity of cancer-associated mutations in p53 with ion-mobility mass spectrometry. Angew. Chem. Int. Ed..

[25] Shelimov KB, Clemmer DE, Hudgins RR, Jarrold MF (1997). Protein structure in vacuo: gas-phase conformations of BPTI and cytochrome *c*. J. Am. Chem. Soc..

[26] Gross DS, Schnier PD, Rodriguez-Cruz SE, Fagerquist CK, Williams ER (1996). Conformations and folding of lysozyme ions in vacuo. Proc. Natl. Acad. Sci. U.S.A..

[27] Valentine SJ, Anderson JG, Ellington AD, Clemmer DE (1997). Disulfide-intact and -reduced lysozyme in the gas phase: conformations and pathways of folding and unfolding. J. Phys. Chem. B.

[28] McLafferty FW, Guan Z, Haupts U, Wood TD, Kelleher NL (1998). Gaseous conformational structures of cytochrome *c*. J. Am. Chem. Soc..

[29] Clemmer DE, Hudgins RR, Jarrold MF (1995). Naked protein conformations: cytochrome *c* in the gas phase. J. Am. Chem. Soc..

[30] Beveridge R, Migas LG, Payne KAP, Scrutton NS, Leys D, Barran PE (2016). Mass spectrometry locates local and allosteric conformational changes that occur on cofactor binding. Nat. Commun..

[31] Pacholarz KJ, Porrini M, Garlish RA, Burnley RJ, Taylor RJ, Henry AJ, Barran PE (2014). Dynamics of intact immunoglobulin G explored by drift-tube ion-mobility mass spectrometry and molecular modeling. Angew. Chem. Int. Ed..

[32] Brocca S, Testa L, Sobott F, Samalikova M, Natalello A, Papaleo E, Lotti M, De Gioia L, Doglia SM, Alberghina L, Grandori R (2011). Compaction properties of an intrinsically disordered protein: Sic1 and its kinase-inhibitor domain. Biophys. J..

[33] Müller-Späth S, Soranno A, Hirschfeld V, Hofmann H, Rüegger S, Reymond L, Nettels D, Schuler B (2010). Charge interactions can dominate the dimensions of intrinsically disordered proteins. Proc. Natl. Acad. Sci. U.S.A..

[34] Breuker K, McLafferty FW (2008). Stepwise evolution of protein native structure with electrospray into the gas phase, 10(-12) to 10(2) s. Proc. Natl. Acad. Sci. U.S.A..

[35] Hogan CJ, Ruotolo BT, Robinson CV, Fernandez De La Mora J (2011). Tandem differential mobility analysis-mass spectrometry reveals partial gas-phase collapse of the GroEL complex. J. Phys. Chem. B.

[36] Kaddis CS, Lomeli SH, Yin S, Berhane B, Apostol MI, Kickhoefer VA, Rome LH, Loo JA (2007). Sizing large proteins and protein complexes by electrospray ionization mass spectrometry and ion mobility. J. Am. Soc. Mass Spectrom..

[37] Mehmood S, Marcoux J, Hopper JTS, Allison M, Liko I, Borysik AJ, Robinson CV, Allison TM, Borysik J (2014). Charge reduction stabilizes intact membrane protein complexes for mass spectrometry. Charge reduction stabilizes intact membrane protein complexes for mass spectrometry. J. Am. Chem. Soc..

[38] Freeke J, Robinson CV, Ruotolo BT (2010). Residual counter ions can stabilize a large protein complex in the gas phase. Int. J. Mass Spectrom..

[39] Hall Z, Politis A, Bush MF, Smith LJ, Robinson CV (2012). Charge state-dependent compaction and dissociation of protein complexes: insights from ion mobility and molecular dynamics. J. Am. Chem. Soc..

[40] McLuckey SA, Stephenson JL (1999). Ion/ion chemistry of high-mass multiply charged ions. Mass Spectrom. Rev..

[41] Gunawardena HP, He M, Chrisman PA, Pitteri SJ, Hogan JM, Hodges BDM, McLuckey SA (2005). Electron transfer versus proton transfer in gas-phase ion/ion reactions of polyprotonated peptides. J. Am. Chem. Soc..

[42] Liu J, Gunawardena HP, Huang T-Y, McLuckey SA (2008). Charge-dependent dissociation of insulin cations via ion/ion electron transfer. Int. J. Mass Spectrom..

[43] Liu J, McLuckey SA (2012). Electron transfer dissociation: effects of cation charge state on product partitioning in ion/ion electron transfer to multiply protonated polypeptides. Int. J. Mass Spectrom..

[44] Prentice, B.M., McLuckey, S.A.: Gas-phase ion/ion reactions of peptides and proteins: acid/base, redox, and covalent chemistries. Chem. Commun. (Camb.) **49**(947–965) (2013) 10.1039/c2cc36577d PMC3557538 23257901

[45] Coon JJ, Shabanowitz J, Hunt DF, Syka JEP (2005). Electron transfer dissociation of peptide anions. J. Am. Soc. Mass Spectrom..

[46] Chi A, Huttenhower C, Geer LY, Coon JJ, Syka JEP, Bai DL, Shabanowitz J, Burke DJ, Troyanskaya OG, Hunt DF (2007). Analysis of phosphorylation sites on proteins from *Saccharomyces cerevisiae* by electron transfer dissociation (ETD) mass spectrometry. Proc. Natl. Acad. Sci. U.S.A..

[47] Jhingree JR, Beveridge R, Dickinson ER, Williams JP, Brown JM, Bellina B, Barran PE (2016). Electron transfer with no dissociation ion mobility-mass spectrometry (ETnoD IM-MS). The effect of charge reduction on protein conformation. Int. J. Mass Spectrom..

[48] Kassell B, Radicevic M, Ansfield MJ, Laskowski M (1965). The basic trypsin inhibitor of bovine pancreas IV. The linear sequence of the 58 amino acids. Biochem. Biophys. Res. Commun..

[49] Kassell B, Laskowski M (1965). The basic trypsin inhibitor of bovine pancreas. V. The disulfide linkages. Biochem. Biophys. Res. Commun..

[50] Bhat M, Dar T, Singh LR (2016). Milk Proteins – from structure to biological properties and health aspects. Intech: Croatia.

[51] Tompa P (2005). The interplay between structure and function in intrinsically unstructured proteins. FEBS Lett..

[52] Perticaroli S, Nickels JD, Ehlers G, Mamontov E, Sokolov AP (2014). Dynamics and rigidity in an intrinsically disordered protein, beta-casein. J. Phys. Chem. B.

[53] Testa L, Brocca S, Santambrogio C, D'Urzo A, Habchi J, Longhi S, Uversky VN, Grandori R (2013). Extracting structural information from charge ionization mass spectrometry. Extracting structural information from charge-state distributions of intrinsically disordered proteins by nondenaturing electrospray-ionization mass spectrometry. Intrinsically Disord. Proteins.

[54] Jurneczko E, Cruickshank F, Porrini M, Nikolova P, Campuzano IDG, Morris M, Barran PE (2012). Intrinsic disorder in proteins: a challenge for (un)structural biology met by ion mobility-mass spectrometry. Biochem. Soc. Trans..

[55] Dickinson ER, Jurneczko E, Pacholarz KJ, Clarke DJ, Reeves M, Ball KL, Hupp T, Campopiano D, Nikolova PV, Barran PE (2015). Insights into the conformations of three structurally diverse proteins: cytochrome *c*, p53, and MDM2, provided by variable-temperature ion mobility mass spectrometry. Anal. Chem..

[56] Saikusa K, Kuwabara N, Kokabu Y, Inoue Y, Sato M, Iwasaki H, Shimizu T, Ikeguchi M, Akashi S (2013). Characterization of an intrinsically disordered protein complex of Swi5-Sfr1 by ion mobility mass spectrometry and small-angle X-ray scattering. Analyst.

[57] Williams, J.P., Brown, J.M., Campuzano, I., Sadler, P.J.: Identifying drug metallation sites on peptides using electron transfer dissociation (ETD), collision-induced dissociation (CID) and ion mobility-mass spectrometry (IM-MS). Chem. Commun. (Camb) **46**(5458–5460) (2010) 10.1039/c0cc00358a 20505878

[58] Pringle SD, Giles K, Wildgoose JL, Williams JP, Slade SE, Thalassinos K, Bateman RH, Bowers MT, Scrivens JH (2007). An investigation of the mobility separation of some peptide and protein ions using a new hybrid quadrupole/traveling wave IMS/oa-ToF instrument. Int. J. Mass Spectrom..

[59] Zhong Y, Hyung S-J, Ruotolo BT (2011). Characterizing the resolution and accuracy of a second-generation traveling-wave ion mobility separator for biomolecular ions. Analyst.

[60] Wallace A (2010). A high-resolution ion mobility mass spectrometry platform for breakthrough discoveries in life science research and the pharmaceutical industry. Am. Lab..

[61] Wlodawer A, Walter J, Huber R, Sjölin L (1984). Structure of bovine pancreatic trypsin inhibitor. Results of joint neutron and X-ray refinement of crystal form. II. J. Mol. Biol..

[62] Mack E (1925). Average cross-sectional areas of molecules by gaseous diffusion methods. J. Am. Chem. Soc..

[63] von Helden G, Hsu MT, Gotts N, Bowers MT (1993). Carbon cluster cations with up to 84 atoms: structures, formation mechanism, and reactivity. J. Phys. Chem..

[64] Massonnet P, Upert G, Smargiasso N, Gilles N, Quinton L, De Pauw E (2015). Combined use of ion mobility and collision-induced dissociation to investigate the opening of disulfide bridges by electron-transfer dissociation in peptides bearing two disulfide bonds. Anal. Chem..

[65] Zhurov KO, Fornelli L, Wodrich MD, Laskay ÜA, Tsybin YO (2013). Principles of electron capture and transfer dissociation mass spectrometry applied to peptide and protein structure analysis. Chem. Soc. Rev..

[66] Echterbille J, Quinton L, Gilles N, De Pauw E (2013). Ion mobility mass spectrometry as a potential tool to assign disulfide bonds arrangements in peptides with multiple disulfide bridges. Anal. Chem..

[67] Mitchell Wells J, McLuckey SA (2005). Collision‐induced dissociation (CID) of peptides and proteins. Methods Enzymol..

[68] Beveridge R, Chappuis Q, Macphee C, Barran P (2013). Mass spectrometry methods for intrinsically disordered proteins. Analyst.

[69] Smyth E, Clegg RA, Holt C (2004). A biological perspective on the structure and function of caseins and casein micelles. Int. J. Dairy Technol..

[70] Salaün F, Mietton B, Gaucheron F (2005). Buffering capacity of dairy products. Int. Dairy J..

[71] Cordeschi M, Di Paola L, Marrelli L, Maschietti M (2003). Net proton charge of β- and κ-casein in concentrated aqueous electrolyte solutions. Biophys. Chem..

[72] Fernandez De La Mora J (2000). Electrospray ionization of large multiply charged species proceeds via Dole’s charged residue mechanism. Anal. Chim. Acta.

[73] Loo RRO, Loo JA (2016). Salt bridge rearrangement (SaBRe) explains the dissociation behavior of noncovalent complexes. J. Am. Soc. Mass Spectrom..

[74] Zhang Z, Vachet RW (2016). Gas-phase protein salt bridge stabilities from collisional activation and electron transfer dissociation. Int. J. Mass Spectrom..

[75] Valentine SJ, Counterman AE, Clemmer DE (1997). Conformer-dependent proton-transfer reactions of ubiquitin ions. J. Am. Soc. Mass. Spectrom..

