# Supplementary material for: Charge Mediated Compaction and Rearrangement of Gas-Phase Proteins: A Case Study Considering Two Proteins at Opposing Ends of the Structure-Disorder Continuum
Source: J Am Soc Mass Spectrom. 2017 Jun 5;28(7):1450–61. doi: 10.1007/s13361-017-1692-1 (PMC5486678; doi:10.1007/s13361-017-1692-1)
Supplement: Supplementary file 1 — (DOCX 633 kb) [file 13361_2017_1692_MOESM1_ESM.docx]

Supporting Information

**Charge mediated compaction and rearrangement of gas phase proteins: A Case Study Considering Two Proteins at Opposing Ends of the Structure-Disorder Continuum**

Jacquelyn R. Jhingree, Bruno Bellina, Kamila J. Pacholarz and Perdita E. Barran

Manchester Institute of Biotechnology, University of Manchester, 131 Princess Street, Manchester, M1 7DN. UK.

**Corresponding author**

Email: perdita.barran@manchester.ac.uk

**Figure S1** nanoESI mass spectra of 10 µM bovine pancreatic trypsin inhibitor (BPTI) sprayed from 50 mM ammonium acetate pH 7 and acquired on a Synapt G2 Si travelling wave ion mobility mass spectrometer.


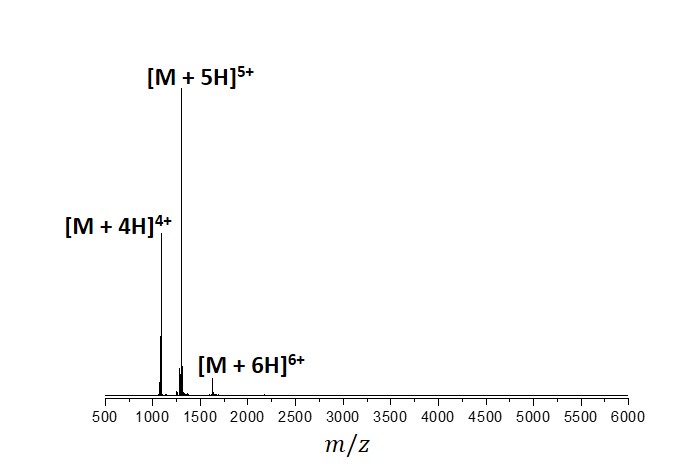


**Figure S2** Isotopic distributions of BPTI obtained by nESI (a,) and ETnoD (b, c). Solid and open circles represent theoretical isotopic distributions for the [M+4H]^4+^ ion (a-c) and radical cations, [M+5H]^4+.^ (b) and [M+6H]^4+..^ respectively.

**
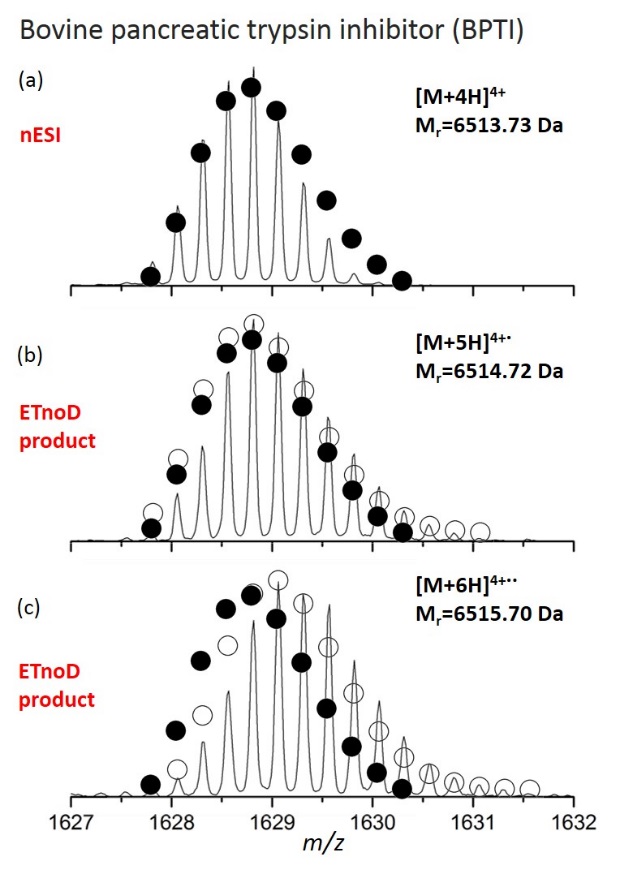
**

**Figure S3** Unfolding of the (a) [M+5H]^5+^ and (b) [M+6H]^6+^ ion of bovine pancreatic trypsin inhibitor (BPTI) upon collisional activation in the trap region of a travelling wave ion mobility mass spectrometer. Each ion is m/z selected in a quadrupole prior to injection into the trap where the collision voltage is incrementally raised (2V-55V) before the ion is mobility separated. The mass spectra to the left of each figure show the zoomed in region around each precusor ion depicting dehydrated forms to the left of each precusor.

1. **
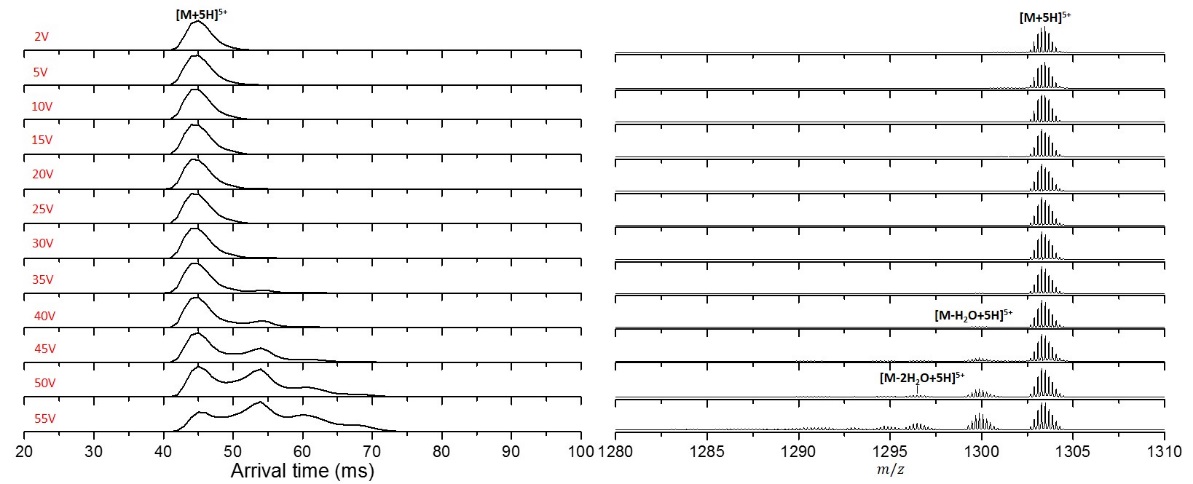
**
2. **
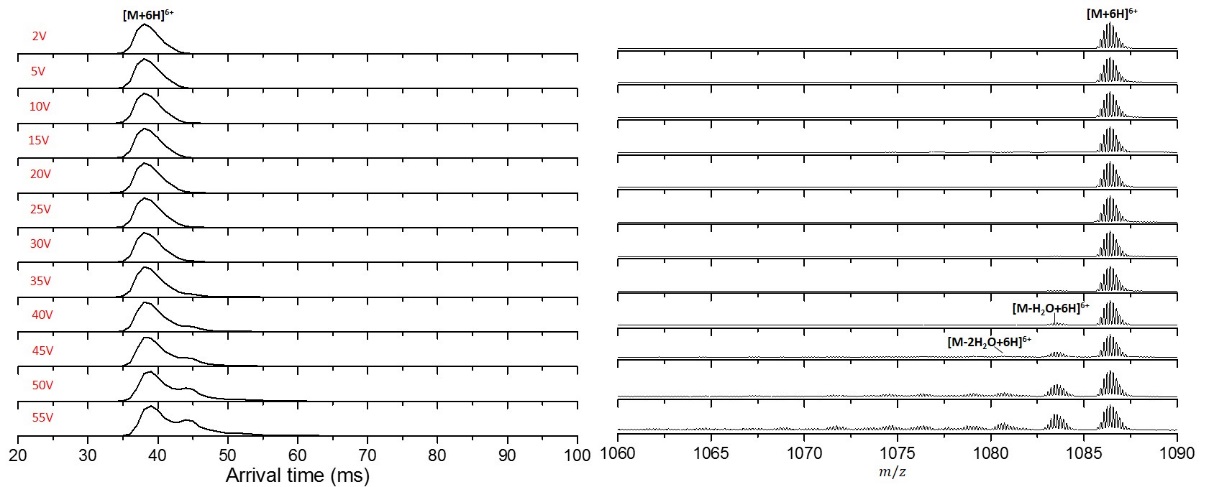
**

**Figure S4** nanoESI mass spectra of 10 µM beta casein sprayed from 50 mM ammonium acetate pH 7 and acquired on a Synapt G2 Si travelling wave ion mobility mass spectrometer.

**
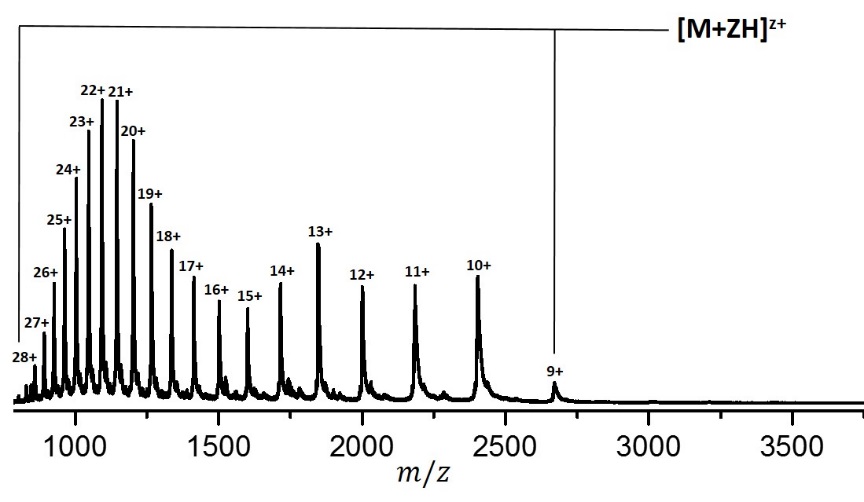
**

**Figure S5** Collision cross section distributions (*^TW^*$CCS_{N_{2}\to He}$) obtained on a Synapt G2 Si travelling wave ion mobility mass spectrometry. Measurements were done in nitrogen and calibrated against cytochrome c and myglobin sprayed under denatured conditions. Literature cross sections for the latter proteins measured in helium were taken from Clemmer’s database. 10 µM beta casein was sprayed from 50 mM ammonium acetate.


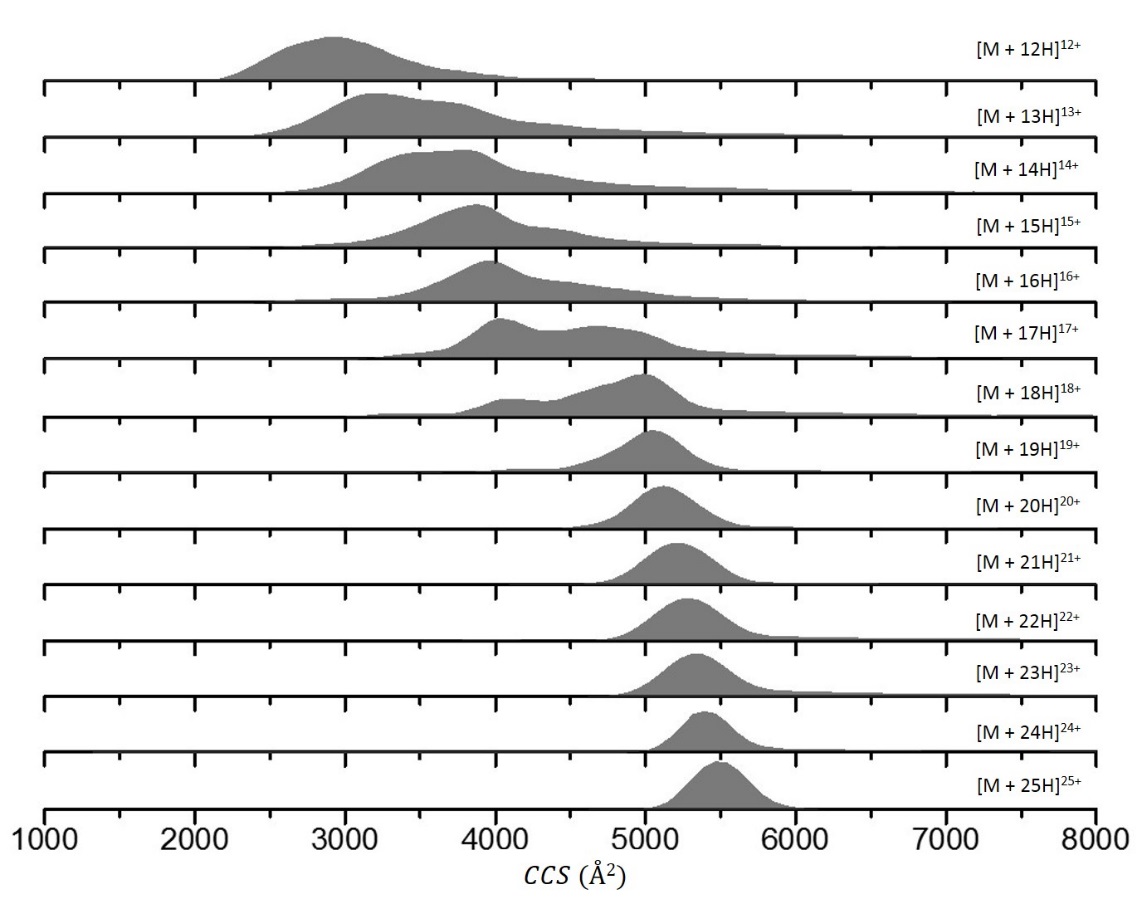


**Table S1** Table depicting the travellinging wave collision cross sections (*^TW^*$CCS_{N_{2}\to He}$) of bovine pancreatic trypsin inhibitor (BPTI) before and after exposure to radical anions of 1,3-dicyanobenzene. Solutions were sprayed from 50 mM ammonium acetate, pH 7. The values stated are the mean of two replicate measurements with standard deviations given.

| *nESI generated*  *ion, ^TW^*$CCS_{N_{2}\to He}$ *(Å^2^)* | | *Post exposure, ^TW^*$CCS_{N_{2}\to He}$ *(Å^2^)* | | |
| --- | --- | --- | --- | --- |
|  |  | 6+ | 5+ | 4+ |
| 6+ | 900 | 915±7 | 808±12 | 800 |
| 5+ | 800 |  | 817 | 800 |
| 4+ | 793 |  |  | 796 |

**Table S2** Table depicting the travellinging wave collision cross sections (*^TW^*$CCS_{N_{2}\to He}$) of beta casein before and after exposure to radical anions of 1,3-dicyanobenzene. Solutions were sprayed from 50 mM ammonium acetate, pH 7. The values stated are the mean of two replicate measurements with standard deviations given.

| *nESI generated*  *ion, ^TW^*$CCS_{N_{2}\to He}$ *(Å^2^)* | | *Post exposure, ^TW^*$CCS_{N_{2}\to He}$ *(Å^2^)* | | | | | | | | | | | | | | |
| --- | --- | --- | --- | --- | --- | --- | --- | --- | --- | --- | --- | --- | --- | --- | --- | --- |
|  |  | 25+ | 24+ | 23+ | 22+ | 21+ | 20+ | 19+ | 18+ | 17+ | 16+ | 15+ | 14+ | 13+ | 12+ | 11+ |
| 25+ | 5638±113 | 5598±57 | 5567±1 | 5556±1 | 5490±50 | 5375±50 |  |  |  |  |  |  |  |  |  |  |
| 24+ | 5528±55 |  | 5528±54 | 5519±52 | 5490±50 | 5409±95 | 5376±45 |  |  |  |  |  |  |  |  |  |
| 23+ | 5554±102 |  |  | 5445±52 | 5420±50 | 5409±1 | 5376±45 | 5198±86 |  |  |  |  |  |  |  |  |
| 22+ | 5455±1 |  |  |  | 5385±1 | 5341±1 | 5343±1 | 5259±1 | 5097±163 |  |  |  |  |  |  |  |
| 21+ | 5375±48 |  |  |  |  | 5341±1 | 5311±45 | 5259±1 | 5213±82 | 5168±39 |  |  |  |  |  |  |
|  |  |  |  |  |  |  |  |  | 3276±123 |  |  |  |  |  |  |  |
| 20+ | 5279±1 |  |  |  |  |  | 5279±1 | 5259±1 | 5155±1 | 5087±1 |  |  |  |  |  |  |
| 19+ | 5198±1 |  |  |  |  |  |  | 5228±43 | 5184±41 | 4923±154 |  |  |  |  |  |  |
|  |  |  |  |  |  |  |  |  | 4808±1 |  |  |  |  |  |  |  |
| 18+ | 4230±1 |  |  |  |  |  |  |  | 4490±41 | 4322±77 | 4247±36 |  |  |  |  |  |
|  | 5097±1 |  |  |  |  |  |  |  | 4779±41 | 4950±116 | 4761±36 |  |  |  |  |  |
|  |  |  |  |  |  |  |  |  | 5184±41 |  |  |  |  |  |  |  |
| 17+ | 4186±39 |  |  |  |  |  |  |  |  | 4240±39 | 4170±73 |  |  |  |  |  |
|  | 4977±77 |  |  |  |  |  |  |  |  | 5005±39 | 4838±1 |  |  |  |  |  |
| 16+ | 4093±36 |  |  |  |  |  |  |  |  |  | 4119±1 | 4029±34 | 3993±21 |  |  |  |
|  | 4658±36 |  |  |  |  |  |  |  |  |  |  |  |  |  |  |  |
| 15+ | 3957±68 |  |  |  |  |  |  |  |  |  |  | 4029±34 | 3895±32 |  |  |  |
| 14+ | 3940±32 |  |  |  |  |  |  |  |  |  |  |  | 3873±1 | 3763±59 |  |  |
| 13+ | 3345±59 |  |  |  |  |  |  |  |  |  |  |  |  | 3533±89 | 3358±2 |  |
|  | 3804±1 |  |  |  |  |  |  |  |  |  |  |  |  | 4472 |  |  |
| 12+ | 2991±82 |  |  |  |  |  |  |  |  |  |  |  |  |  | 3072±53 | 2643±1 |
